# The effect of advanced maternal age and gestational weight gain on newborns

**DOI:** 10.1590/1806-9282.20240961

**Published:** 2025-03-31

**Authors:** Ayse Yazan Arslan, Cüneyt Ardic, Beril Kara Esen

**Affiliations:** 1 Recep Tayyip Erdogan University, Faculty of Medicine, Department of Family Medicine – Rize, Türkiye.; 2 Esenler District Health Directorate, Department of Public Health – İstanbul, Türkiye.

**Keywords:** Maternal age, Gestational weight gain, Large for gestational age

## Abstract

**INTRODUCTION:**

Advanced maternal age is defined as the mother being ≥35 years of age at the time of birth. Gestational weight gain is the difference between the weight measurements of the pregnant woman at prenatal follow-up just before birth and at pre-conception or at the beginning of the first trimester. In our study, we examined the effects of maternal age and gestational weight gain on neonatal outcomes.

**METHODS:**

This study was conducted, in the Rize province between April 2022 and January 2023, on pregnant women registered in seven Family Health Centers in the last 5 years and their resulting newborns. In total, this study analyzed the data of 642 pregnant women and their 642 newborns. Data records in the study are pregnant-puerperal follow-up form, newborn-child follow-up form, and family medicine information system.

**RESULTS:**

The probability of newborns of mothers with excessive gestational weight gain being large-for-gestational-age was found to be 1.81 times higher in univariate analysis and 1.72 times higher in multivariate analysis. While the effect of gestational weight gain status on birth weight Z-score was significant, gestational age had no significant effect (p=0.001 and p=0.136, respectively).

**CONCLUSION:**

In this period, when obesity, which is a disease of our age, is widespread and the gestational age has moved to older ages, there is a need for more extensive research on this subject in future studies.

## INTRODUCTION

Advanced maternal age (AMA) is defined as the mother being ≥35 years of age at the time of birth^1,2^. When the birth statistics of the Turkish Statistical Institute are examined, the birth rate for those aged 35 years and over was 14.27% in 2015, which increased to 15.89% in 2021^3^. The Organization for Economic Co-operation and Development (OECD) reported that since 2000, while fertility rates decreased for women under 30 years of age, the reverse was true for those aged ≥30 years^4^. Literature search revealed that AMA is associated with numerous adverse pregnancy and neonatal outcomes^5–7^.

Gestational weight gain (GWG) is the difference between the weight measurements of the pregnant woman at prenatal follow-up just before birth and at pre-conception or at the beginning of the first trimester^8^. It is known that excessive weight gained during pregnancy or, conversely, inadequate weight has adverse consequences for both the newborn and the mother. For example, weight gain above recommendations has been associated with increased macrosomia, large-for-gestational-age newborns (LGA), and cesarean delivery^9^.

This study is the first in Turkey in which Z-score measurements of birth weight, birth length, and birth head circumference were calculated using Intergrowth 21 standards of newborns of mothers whose GWG was categorized according to IOM and age group.

In the thesis study conducted by Akgün in 2013, the effects of maternal body mass index (BMI) and GWG on neonatal outcomes were examined, similar to our study. However, in our study, GWG categories were also subcategorized according to age and evaluated as <35 years of age and ≥35 years of age^10^.

The aim of this study was to classify the pre-pregnancy BMIs of pregnant women according to the WHO criteria, evaluate their gestational weight gain according to the pregnancy weight gain guide recommendations published by IOM in 2009, and examine the effects of maternal age and gestational weight gain on newborn outcomes.

## METHODS

### Data collection

This study was conducted, in the Rize province between April 2022 and January 2023, on pregnant women registered in 34 Family Medicine Units (FMU) of 7 Family Health Centers (FHC) in the last 5 years and their resulting newborns.

In total, 320 pregnant women aged ≥35 years were included in the study. Data pertaining to 322 pregnant women under the age of 35 years were collected in a similar fashion. In total, this study analyzed the data of 642 pregnant women and their 642 newborns.

### Inclusion criteria

Pregnant women aged 18 years and over, whose birth week was known according to the last menstrual period, were included in the study. In addition, pregnant women who applied for the first follow-up before the 8th week of gestation, whose height and weight were measured by the nurse on duty at the FHC, and who were followed up at least four times in total or at least once in each trimester were included. In cases where the same person had different pregnancies within a 5-year period, the last pregnancy period and information were included in the study.

### Exclusion criteria

Pregnant women with multiple pregnancy status, pre-pregnancy hypertension, gestational hypertension or preeclampsia, pre-gestational diabetes mellitus or gestational diabetes mellitus, thromboembolic complications during pregnancy, a history of polyhydramnios or oligohydramnios during pregnancy, hypothyroidism, and chronic kidney disease were not included in the study.

Data records of pregnant women and newborns included in the study

1. Pregnant-puerperal follow-up form (PPFF)
2. Newborn-child follow-up form (NCFF)
3. Family Medicine Information System (FMIS)

The PPFF begins to be recorded as of the day the person applies for the first pregnancy monitoring by the nurse in the FMU to which the person is affiliated. Personal information such as the pregnant woman's date of birth, marital status, education level, smoking status, and employment status are provided here. Obstetric history, last menstrual period in the current pregnancy, first follow-up week, weight and height of the pregnant woman measured by the nurse or midwife at the first and last follow-up, gestational week at the end of pregnancy, birth time according to week, and delivery method are among the data obtained here.

The NCFF will be filled out by the nurse at the FHC to which the newborn is brought for follow-up in the first week after birth. Included in the form are the newborn's gender, mode of birth, birth weight, birth length, head circumference at birth, and congenital malformation status, and the results of the newborn data were analyzed in the study.

### Pre-pregnancy maternal BMI analysis

BMI is calculated by dividing a person's weight in kilograms by the square of their height in meters (kg/m^2^)^11^. This measure is independent of age, race, ethnicity, parity, and muscle mass in adults.

### Gestational weight gain

It has been reported in the literature that there is a strong correlation between the pre-pregnancy period and the first 8 weeks of pregnancy in terms of maternal BMIs^12^. In order to minimize the impact of the limitation on the study, pregnant women who applied for the first follow-up before the 8th week of pregnancy were included.

In our study, recommended total weight gain ranges in singleton pregnancies according to the BMI category calculated based on the WHO BMI classification in the 2009 IOM criteria were used. The recommended total weight gain ranges are 12.5–18 kg for underweight women, 11.5–16 kg for normal-weight women, 7–11.5 kg for overweight women, and 5–9 kg for obese women.

### Effect on neonatal outcomes

Newborns are classified as preterm, term, and postterm according to the week of gestation they are born. Preterm birth is used to describe newborns born alive before completing the 37th week of gestation. Postterm birth refers to the birth of live newborns after the 42nd week of gestation^13^. Birth weight percentile measurement of newborns was calculated using International Fetal and Newborn Growth Consortium for the 21st Century (INTERGROWTH-21st) standards specific to gestational age and gender^14^. Newborns were categorized as small-for-age (SGA) newborns, gestational-age-appropriate (AGA) newborns, and LGA newborns based on the classification of the American Academy of Pediatrics. SGA is defined as birth weight below the 10th percentile for gestational age, and LGA as birth weight above the 90th percentile^15^. Newborns were also classified by birth weight, regardless of gestational age. In our study, weights of 4,000 g and above were classified as macrosomia.

### Z-score measurements of newborns

Birth anthropometers of newborns were determined based on INTERTGROWTH-21st standards. Z-scores of the newborn were evaluated by entering the data into the calculation section on the INTERGROWTH-21st website using gender, birth week, birth weight, height, and head circumference^16^.

### Statistical analysis

The SPSS 21.0 package program was used in the analysis of the study. The suitability of the data for normal distribution was evaluated with the Kolmogorov-Smirnov test and coefficient of variation. Continuous variables were shown with their mean, standard deviation, minimum, and maximum values. Categorical variables are shown with frequency and percentage. The chi-square test or Fisher's exact test was used for categorical variables for comparisons between groups. Assessment of continuous variables was made by Student's t test between the two groups and one-way analysis of variance (ANOVA) test between more than two groups. Binomial logistic regression analysis was performed to evaluate preterm and LGA risk of newborns, and and the data were presented as odds ratios (OR) and 95% confidence intervals (CIs). The results were evaluated at a 95% confidence level, and the significance level was accepted as p<0.05.

## RESULTS

### Demographic characteristics of maternal and infant characters

Among pregnant women aged ≥35 years, the rates of adequate, inadequate, and excessive GWG states were 35, 28, and 37%, respectively. When evaluated according to the GWG, the LGA rate in the newborns of mothers aged ≥35 years with excessive GWG was higher than that of mothers with inadequate and adequate GWG, but this difference was insignificant (Table 1).

**Table 1:** Maternal and infant characteristics and demographics according to maternal age and gestational weight gain categories.

		Inadequate			Adequate			Excessive		
		(n=173)		p	(n=233)		p	(n=236)		p
		<35	≥35		<35	≥35		<35	≥35	
Maternal characteristics		n=84	n=89		n=121	n=112		n=117	n=119	
Parity	Primipara	26 (31)	8 (9)	**<0.001** *	44 (36.4)	13 (11.6)	**<0.001** *	40 (34.2)	11 (9.2)	**<0.001** *
	Primary-Secondary	23 (27.4)	39 (43.8)	**0.031** *	36 (29.8)	46 (41.4)	0.176*	26 (22.2)^a^	53 (44.9)^b^	**0.001** *
Education	High School	25 (29.8)	27 (30.3)		37 (30.6)	29 (26.1)		40 (34.2)^a^	31 (26.3)^a^	
	University	36 (42.9)	23 (25.8)		48 (39.7)	36 (32.4)		51 (43.6)^a^	34 (28.8)^b^	
Smoking status	Smoker	1 (1.2)	5 (5.6)	0.112**	11 (9.1)	2 (1.8)	**0.015** **	8 (6.8)	9 (7.6)	0.829*
Infant characteristics		n=84	n=89		n=121	n=112		n=117	n=119	
Gender	Female	38 (45.2)	47 (52.8)	0.319*	61 (50.4)	51 (45.5)	0.457*	50 (42.7)	57 (47.9)	0.426*
Birth Time	Preterm	14 (16.7)	23 (25.8)	0.141*	14 (107)	19 (17)	0.238*	12 (10.3)	17 (14.3)	0.346*
	SGA	3 (3.6)	3 (3.4)	0.533**	3 (2.5)	4 (3.6)	0.213**	3 (2.6)	5 (4.2)	0.849**
Fetal	AGA	70 (83.3)	69 (77.5)		103 (85.1)	85 (75.9)		83 (70.9)	84 (70.6)	
Growth	LGA	11 (13.1)	17 (19.1)		15 (12.4)	23 (20.5)		31 (26.5)	30 (25.2)	
	<2500	0	6 (6.7)	0.046**	4 (3.3)	2 (1.8)	0.352**	1 (0.9)	2 (1.7)	0.089**
Birth	2500-4000	80 (95.2)	79 (88.8)		110 (90.9)	98 (87.5)		96 (82.1)	107 (89.9)	
Weight	>4000	4 (4.8)	4 (4.5)		7 (5.8)	12 (10.7)		20 (17.1)	10 (8.4)	

* Chi-squared test,

** Fisher's exact test.

GWG: gestational weight gain; SGA: small-for-age; AGA: gestational-age-appropriate; LGA: large-for-gestational-age. Same superscript letter denotes a subset of categories whose column value do not differ significantly form each other at the 0.05 level.

### Effect of maternal age and gestational weight gain on newborn outcomes

The probability of newborns of mothers with excessive GWG being LGA was found to be 1.81 times higher in univariate analysis (crude OR 1.81 [95%CI 1.15–2.85]) and 1.72 times higher in multivariate analysis (adjusted OR 1.72 [95%CI 1.08–2.74]) (Table 2).

**Table 2:** Assessment of maternal age and gestational weight gain status on neonatal outcomes.

		Preterm			LGA		
		n (%)	Crude	Adjusted^a^	n (%)	Crude	Adjusted^b^
			OR (95% CI)	OR (95% CI)		OR (95% CI)	OR (95% CI)
**Variables**							
Age	**<35**	40 (40.4)	Ref. (1)	Ref. (1)	57 (44.9)	Ref. (1)	Ref. (1)
	≥**35**	59 (59.6)	**1.6 (1.04**–**2.47)**	1.34 (0.82–2.20)	70 (55.1)	1.32 (0.89–1.95)	1.27 (0.81–1.98)
	Adequate	37 (37.4)	Ref. (1)	Ref. (1)	28 (22)	Ref. (1)	Ref. (1)
GWG	Inadequate	33 (33.3)	1.64 (0.98–2.75)	1.65 (0.98–2.8)	38 (29.9)	0.997 (0.58–1.70)	0.94 (0.55–1.62)
	Excessive	29 (29.3)	0.85 (0.5–1.44)	0.80 (0.47–1.38)	61 (48)	**1.81 (1.15–2.85)**	**1.72 (1.08–2.74)**

a Nagelkerke R^2^: 0.047;

b Nagelkerke R^2^: 0.057.

Other variables included in the models: Gravida, newborn gender, and smoking status. LGA: large-for-gestational-age; OR: odds ratio; CI: confidence interval; GWG: gestational weight gain.

### Relationship between newborn Z-scores according to maternal age and gestational weight gain status

Both the GWG status of the pregnant women and the gestational age significantly affected the birth length Z-score (p=0.004 and p<0.001, respectivel).

## DISCUSSION

### Demographic characteristics

In our study, it was determined that the distribution of pregnant women into GWG categories according to age was similar among themselves. In the study conducted by Xiao et al., in pregnant women aged ≥35 years, the rates of inadequate and adequate weight were, respectively, 18.3 and 46.3%^17^. Chinese BMI categories have different value ranges than those of WHO BMI categories^18^. We can explain the high rate of people with adequate weight gain according to BMI by using BMI values categorized according to their own race.

### Neonatal outcomes

In our study, we evaluated the relationship between neonatal outcomes, such as LGA and preterm birth with GWG, and maternal age. Consistent with the literature, we found that excessive GWG significantly increased the risk of LGA. In a meta-analysis consisting of selected studies similar to ours, it was reported that pregnant women with excessive GWG had a higher risk of LGA (OR 1.85 [95%CI 1.76–1.95]). In the same study, the risk of LGA was also found to be lower in pregnant women with inadequate GWG (OR 0.59 [95%CI 0.55–0.64])^9^.

In our study, the relationship between neonatal Z-score measurements and GWG was evaluated. As GWG increased, birth weight Z-score and birth length Z-score also increased. A similar study by Bauserman et al. reported no significant relationship between GWG and newborn birth weight Z-score measurement and newborn birth length Z-score^19^. In this study, the last weight measurement of the mothers was made at the 32nd week, and the potential effects of GWG in the last weeks of pregnancy could not be observed sufficiently.

**Table 3:** Newborn birth weight Z-score, birth length Z-score, and birth head circumference Z-score measurements according to age group and gestational weight gain status.

		Birth weight Z-score	p-value	Birth length Z-score	p-value	Birth head circumference Z-score	p-value
Age	<35	0.42±0.92	0.136*	0.38±089	**<0.001** *	1.01±0.96	0.326*
	≥35	0.53±1		0.64±0.92		1.08±1.04	
GWG	Inadequate (n=173)	0.31±0.96^a^	**0.001** **	0.39±0.92^a^	**0.004** **	0.97±1.03	0.303**
	Adequate (n=233)	0.41±0.92^a^		0.44±0.88^a^		1.02±1	
	Excessive (n=236)	0.66±0.97^b^		0.66±0.93^b^		1.12±0.98	

* Student's t-test,

** one-way ANOVA.

GWG: gestational weight gain. The same superscript letter denotes a subset of GWG categories that do not differ significantly from each other at the 0.05 level.

In our study, many factors that affect weight gain during pregnancy, such as gestational diabetes mellitus, oligohydramnios, and polyhydramnios, were excluded in order to reveal the effect of maternal age. Since pregnancy follow-ups may also have an effect on weight gain, the study was completed with pregnant women who came for follow-up at least four times. The mother's smoking status, which has a proven effect on preterm birth, was also evaluated in our study and was included in the model in the regression.

Our study has several limitations. The study is single-center and the sample size is relatively small. Multi-center studies with a larger number of participants are needed. Some factors that could affect the results, such as the mother's nutrition and supplement intake, and the socioeconomic level of the family, were not taken into account in this retrospectively designed study.

## CONCLUSION

In our study, we determined that inadequate weight gain and AMA negatively affect neonatal outcomes. In addition, we showed that excessive weight gain, especially during pregnancy, results in LGA in the newborn. In this period, when obesity, which is a disease of our age, is widespread and the gestational age has moved to older ages, there is a need for more extensive research on this subject in future studies.

## References

[1] Cunningham FG, Leveno KJ (1995). Childbearing among older women — the message is cautiously optimistic. N Engl J Med.

[2] ACOG (2022). Pregnancy at Age 35 Years or Older.

[3] Turkish Statistical Institute (2022). TURKSTAT Birth Statistics 2021.

[4] OECD Family Database (2022). SF2.3: Age of mothers at childbirth and age-specific fertility.

[5] Correa-De-Araujo R, Yoon SS (2021). Clinical outcomes in high-risk pregnancies due to advanced maternal age. J Women's Heal.

[6] Bayrampour H, Heaman M (2010). Advanced maternal age and the risk of cesarean birth: a systematic review. Birth.

[7] Fitzpatrick KE, Tuffnell D, Kurinczuk JJ, Knight M (2017). Pregnancy at very advanced maternal age: a UK population-based cohort study. BJOG.

[8] Rasmussen KM, Yaktine AL, Institute of Medicine (US) and National Research Council (US) Committee to Reexamine IOM Pregnancy Weight Guidelines (2009). Weight gain during pregnancy: reexamining the guidelines.

[9] Goldstein RF, Abell SK, Ranasinha S, Misso M, Boyle JA, Black MH (2017). Association of gestational weight gain with maternal and infant outcomes: a systematic review and meta-analysis. JAMA.

[10] Akgün N (2013). The relationship between maternal body mass index and body weight gain during pregnancy and perinatal outcomes.

[11] Kominiarek MA, Peaceman AM (2017). Gestational weight gain. Am J Obstet Gynecol.

[12] Hu G, Tian H, Zhang F, Liu H, Zhang C, Zhang S (2012). Tianjin Gestational Diabetes Mellitus Prevention Program: study design, methods, and 1-year interim report on the feasibility of lifestyle intervention program. Diabetes Res Clin Pract.

[13] ACOG (2013). Obstet Gynecol.

[14] Villar J, Cheikh Ismail L, Victora CG, Ohuma EO, Bertino E, Altman DG (2014). International standards for newborn weight, length, and head circumference by gestational age and sex: the Newborn Cross-Sectional Study of the INTERGROWTH-21st Project. Lancet.

[15] Philip AGS (2003). Historical perspectives classification by birthweight and gestational age. Neoreviews.

[16] Intergrowth 21 Newborn size. ManualEntry.

[17] Xiao L, Ding G, Vinturache A, Xu J, Ding Y, Guo J (2017). Associations of maternal pre-pregnancy body mass index and gestational weight gain with birth outcomes in Shanghai, China. Sci Rep.

[18] Zhou B, Coorperative Meta-Analysis Group Of China Obesity Task Force (2002). Predictive values of body mass index and waist circumference to risk factors of related diseases in Chinese adult population. Zhonghua Liu Xing Bing Xue Za Zhi.

[19] Bauserman MS, Bann CM, Hambidge KM, Garces AL, Figueroa L, Westcott JL (2021). Gestational weight gain in 4 low- and middle-income countries and associations with birth outcomes: a secondary analysis of the Women First Trial. Am J Clin Nutr.

